# Research site monitoring for compliance with ethics regulatory standards: review of experience from Uganda

**DOI:** 10.1186/1472-6939-14-23

**Published:** 2013-06-05

**Authors:** Joseph Ochieng, Julius Ecuru, Frederick Nakwagala, Paul Kutyabami

**Affiliations:** 1 Department of Anatomy, School of Biomedical Sciences, College of Health Sciences, Makerere University, Mulago Hill, P.O Box 7072, Kampala, Uganda; 2 Uganda National Council for Science and Technology, Plot 6 Kimera Road, P.O Box 6884, Kampala, Ntinda, Uganda; 3 Mulago Hospital, Mulago Hill, P.O Box 7051, Kampala, Uganda; 4 Department of Pharmacy, College of Health Sciences, Makerere University, P.O Box 7072, Kampala, Uganda

**Keywords:** Research, Monitoring, Compliance, Ethics, Uganda

## Abstract

**Background:**

On site monitoring of research is one of the most effective ways to ensure compliance during research conduct. However, it is least carried out primarily for two reasons: presumed high costs both in terms of human resources and finances; and the lack of a clear framework for undertaking site monitoring. In this paper we discuss a model for research site monitoring that may be cost effective and feasible in low resource settings.

**Methods:**

This was a retrospective review of research site monitoring reports covering a period of four years.

**Results:**

The monitoring was conducted by the Uganda National Council for Science and Technology, the National Drug Authority and the National HIV/AIDS Research and Ethics Committee over the period 2007 to 2010.

The monitoring team was usually three members comprising of two experts in research ethics and an assistant. A total of 28 site monitoring visits covering 40 research projects were reviewed. 25% of the site monitoring reports revealed violation of the regulatory requirement for valid ethical approval. 36% of the site reports showed some instances of informed consent violation, 28% showed violation of the rights and welfare of research participants, 38% revealed that sites did not report SAEs to regulatory authorities and many sites lacked adequate GCP and GCLP. However, most of the sites monitored had adequate facilities to conduct the respective studies and good working practices.

**Conclusion:**

This model employed by the monitoring teams to evaluate research compliance is effective in auditing ethical practice. Compliance monitoring is feasible and affordable in a resource limited setting. Research protocol non compliance is still a major problem in Uganda, and there is need for a pro-active approach to this vice by all stake holders if ethical conduct of research is to be achieved.

## Background

Review of research by an independent research ethics committee (REC) is accepted as a global standard for protecting human research participants [1-9]. Investigators submit their protocols to a research ethics committee for their review and approval prior to initiation of a study. The research ethics committee does initial and continuing review until completion of the study. Continuing review is one of the major challenges of research ethics committees as it involves a number of aspects including annual renewals, amendments to the approved protocols, serious adverse event monitoring, and on site monitoring. The investigators to a large extent play an important role because they have to observe a high level of integrity to comply with ethical standards as well as report any protocol events accurately. However, research ethics committees cannot rely solely on reports by investigators since many times researchers do violate ethical practice [10-13]. RECs have a responsibility to monitor activities of approved studies for ethical conduct as well as adherence to the approved protocol [2].

Research protocol non-compliance occurs in many parts of the world, probably due to ineffective regulatory systems and research participants that are less empowered mainly due to power differences, poverty and ignorance [14-16]. In such settings, protocol non compliance and violations usually occur for approved studies because researchers may not be sufficiently educated about their role in the ethical conduct of research as well as the need to stick to the regulations [10,17]. Hence need for continuing review through research site compliance monitoring in order to minimize such unethical tendencies.

In many countries, a good number of research ethics committees do not monitor approved research protocols for compliance with the required ethical standards and scientific principles. Therefore, there is high likelihood of non-compliance which may compromise the rights and welfare of research participants.

On site monitoring of research is one of the most effective ways to ensure compliance during research conduct. However, it is least carried out primarily for two reasons: presumed high costs both in terms of human resources and finances; and the lack of a clear framework for undertaking site monitoring [18]. In this paper we discuss a model for research site monitoring that may be cost effective and feasible in low resource settings. The model is one used in compliance monitoring by the Uganda National Council for Science and Technology for approved research projects. The discussion is based on a comprehensive review of research compliance monitoring reports carried out on projects approved by the Uganda National Council for Science and Technology (UNCST).

### Objective

The objective of this paper is to demonstrate the feasibility of research compliance monitoring by research ethics committees and evaluate the effectiveness of the monitoring tool in a resource limited setting.

## Methods

We undertook a retrospective review of research site monitoring reports covering a period of four years. A data collection template was developed and utilized in the evaluation of the compliance monitoring framework that is employed by the UNCST and Institutional review Committees.

Although about 50 study projects had been monitored, the review covered only complete reports which were 28 in number comprising 40 studies and includes study sites from both the central, east, west and northern regions of the country. The method used by the compliance monitoring teams included announced or non-announced visits to the study site. The framework employed during the monitoring included reviewing the regulatory documents, informed consent process, study related documentation, participants’ welfare, serious adverse event management and reporting, study related training and working practices at the site.

The monitoring considered both study document review, observations and verbal interviews with study staff and participants.

The documents reviewed included; approved study protocols, REC and UNCST/NDA approvals and their validity, signed informed consent forms, case report forms, data collection forms, valid practicing licenses for clinicians, study related trainings, GCP, GLP and other research ethics trainings, study related meeting minutes, SAEs reports, materials transfer agreements, protocol deviation and protocol violation reports and any other study documents.

Interview where applicable would be held with investigators and or study staff about their training, knowledge of the research regulations, study related meetings, any other studies at the site and how they handled study participants. In addition, a chat with research participants about their knowledge of the study they are participating in, how they are cared for and facilitation like transport refund. In some cases, discussions would be held with the concerned REC.

Observations included assessment of the recruitment areas and surrounding environment, the laboratories, the clinics, dispensing areas, storage facilities for drugs and other study materials, data management areas, number of study staff and record offices. The assessment of the facility evaluated the available space in relation to the approved sample size of the study, number of other studies at the site, frequency of study visits and the congestion that was observed during the monitoring visit. Others included evaluation of the reception for participants, waiting area, care for research participants, how research assistants handle participants including obtaining informed consent, participant waiting time, how and what amounts were re-imbursed for participant transportation and if participants at the study site during lunch were fed.

### Ethical review

Permission to review the site monitoring reports was sought from the Uganda National Council for Science and Technology. No individual identifying information or any particular research site was revealed during the records review.

## Results

All the reports were in the custody of the Uganda National council for Science and Technology. The monitoring had been conducted by both the Uganda National Council for Science and Technology, the National Drug Authority (NDA) as well as the National HIV/AIDS Research and Ethics Committee (NARC), and covered the period 2007 to 2010. Most of the research projects monitored included international collaborative studies.

The monitoring team was in most of the cases made up of three members comprising of two experts in research ethics and an assistant. The site monitoring reports covered studies that included clinical trials, observational, behavioral and basic science studies. And the institutions monitored included; academic institutions, hospitals, research organizations and research demographic sites.

All the site monitoring visits were conducted in the period from 2007 to 2010. Eighteen reports (64.28%) of the reviewed reports showed that the sites visits had been conducted by Uganda National Council for Science and Technology (UNCST), 17.86 of the reviewed reports showed that the sites visits had been conducted jointly by the UNCST and the National Drug Authority (NDA), while another 17.86 of the reviewed reports showed that the sites visits had been conducted by the National HIV/AIDS Research Committee (NARC).

### Reviewing of the monitoring reports employing the model used by the monitoring teams

The model used by the monitoring teams looked at seven elements: regulatory issues, site facilities, informed consent process and documentation, participant’s welfare, reporting and management of adverse events, study related training and working practices. These were the core elements which determined compliance with approved protocol and approval stipulations. They are detailed in Table 1 and discussed below:

**Table 1:** Monitoring activities

**Activity**	**Documentation**	**Interview**	**Observation**
1. Regulatory documents	• Approvals: REC, UNCST and NDA	• If approval is valid	• Availability of study related docummnets
	• Case report forms	• Communication with collaborators	
	• Data collection forms		
	• Valid practicing licenses		
	• Study brochures		
2. Site facilities	• Recruitment area	• How many studies being conducted at the site	• Availability and the amount of space compared to the participant population
	• Laboratories	• Approval status for all studies	• Approval letters
	• Clinics,	• Number of staff for the study	• Available staff
	• dispensing areas,		
	• storage facilities for drugs and other study materials,		
	• data management areas,		
	• study staff		
	• Record offices		
3. Informed consent process and documentation	• Signed informed consent forms	• How it is obtained	• Observe the process of obtaing consent
		• Who witnesses	
		• How long it takes	
4. Participant welfare	• Amount approved in consent form	• How much is given	
	• Signature by receipient	• How amount is determined	
		• Who gives out the cash	
		• Ask participant what they get	
5. SAEs management and reporting	• Records of identified SAEs and their management	• How identified	• SAE reports
	• Signed SAE reports	• How managed	
		• Status of the SAE	
6. Study related training	• Training certificates	• When trained	Training certificates
		• Who trained	Ability to perform as trained
		• Importance of the training	
		• Knowledge of regulations and study	
7. Working practices	• Minutes of meetings	• Frequency of meetings	Meeting minutes
	• Communication memos	What is discussed in the meetings	
	• Communication with collaborators	How long the meetings take	
	• Availability of SOPs • Delegation logs	• Explaining of procedures	SOPs at work stations
8. Debriefing		Communicate findings of the monitoring team and get feedback from research team	

### Regulatory documents

Regulatory aspects are issues to do with the administrative management of the research project to facilitate ethical conduct and include aspects of documentation of communications between the research site and regulatory agencies like RECs, UNCST and NDA; communication between research sites and collaborators; as well as communications between the study coordination team and the other study staff. Before any conduct of research in the country, ethical review and approval must be sought from a competent and independent REC that is recognized by the UNCST and this is followed by clearance by the UNCST.

In this review, 25% of the site monitoring reports revealed expired REC and or UNCST approvals which were not renewed yet studies activities were still ongoing. The regulation stipulates that research can be conducted only and whenever there is a valid REC approval.

In addition, 25% of the site reports revealed either conduct of unapproved research or implementing changes in the protocol without review and approval by the concerned RECs, UNCST and or NDA. Any change to the approved protocol can only be implemented after approval by an IRC unless it is done to protect the participants from eminent harm. Two sites in which were conducted four studies did not have a regulatory file at the sites. In such a situation it would be difficult for the monitoring team to ascertain if the studies had valid approvals as well as other aspects related to administration of the study.

Other regulatory issues included absence of Materials Transfer Agreements for exported Human Biological Materials. In Uganda such materials can only be ethically exported using a permit from the UNCST and based on a valid Materials Transfer agreement between the local and collaborating institutions. Some studies had been terminated but no evident in form of documentation existed at the site or any communication to the regulatory agencies.

Proper filing of study related documents was lacking in many instances and even in situations where quick references would be needed like for SOPs, it was only the electronic copies of such documents available.

One site had a principal investigator who was a foreign national that happened to be the team leader of study activities without being part of the approved protocol and was not registered with any regulatory agency. In addition a number of other protocol violations and deviations were not reported to the concerned RECs.

### Site facilities

Most of the sites monitored had adequate facilities to conduct the respective studies. However, a good number of sites had issues like congestion at recruitment stations, pharmacies that did not appear organized, limited training to study staff in study related issues and research ethics, non-restricted data storage facilities, poor data quality control mechanisms and inadequate/incomplete filing of study related records.

Others included lack of quality dispensing and labeling of drugs as well as lack of SOPs at the respective duty stations.

### Informed consent process and documentation

Informed consent is an essential requirement of ethical research which demands that a research participants’ rights should be respected. Respect for persons requires that research participants be given the opportunity to make choices about what should be done to them. Informed consent is not just a form but a process of information exchange between the researcher and research participants on the whole research process. Information provided should be adequate, clearly understood by the research participant with decision making capacity and the research participant should voluntarily decide to participate.

During the review, it was observed that 36% of the site reports showed some instances where either consent was not obtained, or there was no adequate documentation to prove that informed consent had been obtained. Common consent violations included incomplete consent forms, for example, some pages missing (5%), using study staff to witness consent for illiterate participants (usually study staff have significant conflict of interest and may not objectively witness consent), (5%); unsigned consent forms which could imply that an investigator may have missed reviewing the consent as required by their own protocol (7.5%); and lack of a separate consent for stored specimen as required by the Ugandan guidelines (20%). Five percent of the studies had not obtained informed consent from participants, 10% of the studies continued recruiting participants after expiry of the approved consent form, 7.5% of the studies had consent forms with only a signature page while another 2.5% of the studies reviewed kept participants in the study long after expiry of the time indicted in the consent form. Finally, in a study involving children assent was obtained from children above 13 years only yet it should be children of age 8 and above as per national guidelines.

### Participants’ welfare

During research conduct, participant’s acceptance to participate does not in any way remove their rights from them. Thus protection of participants’ rights and welfare is an obligation for all of those involved in conduct of research. Welfare would require that participants’ needs like meals and refreshments as they wait at the study site, compensation for time due to forgone opportunities and transport costs for study related activities are addressed by the concerned project.

In this review, it was evident that 28% of the reports showed that participants were either not compensated for their time or got inadequate compensation. For example, in some sites, participants waited for long periods without a snack or a meal and were not compensation for time and work lost. In one scenario, participants who normally would not require hospital admission where admitted for more than one week in order to participate in a clinical trial yet no compensation for time lost was done. However, many of the studies that did not adequately facilitate the participants had been approved in that form indicating that the ethical review process had approved the protocol without due consideration for participant welfare.

### SAE: management and reporting

According to the Uganda National Guidelines for conducting research involving humans, all serious adverse events must be reported to the local IRC as soon as possible and in any case no later than seven calendar days of becoming aware of the event. Thereafter, a detailed report of the SAE should be submitted within eight days. The requirement to report adverse events to regulatory authorities does apply to events that are observed among participants in which a health related intervention is being administered. In this review, twenty one (21) studies met the requirement to report SAEs. Eight of the 21 one studies (38%) did not report SAEs to regulatory authorities, two of twenty one (9.5%) reported late while six out of 21 (28.5%)reported SAEs not reviewed and signed by the principal investigator.

### Study related training

It is important that study staff should be knowledgeable and adequately informed about the study they are executing in order to be able to perform efficiently and provide information to participants whenever need arises. In this review, many sites lacked adequate GCP and GCLP. In most cases, only a few members of the study staff had had training. Some sites did not train their staff in study related aspects while most of the sites reviewed had staff who had very little knowledge of research ethics and ignorance particularly about the Uganda National Guidelines.

### Working practices

In working practices, the site monitors where interested in aspects that facilitate coordinated execution of the concerned study including effective communication throughout the study team, updates on any new findings about the study, study coordination and how study staff relate to each other.

Most of the sites reported good working practices like regular study related meetings some as frequent as daily updates, weekly or bi-monthly meetings.

However, some sites almost had no work related meetings which would mean study coordination was defective. Worse of were the sites where the principal investigators were too busy to monitor the conduct of the study on a regular basis and guide their staff, hence the name absentee PIs.

### Debriefing

At the end of a monitoring visit, the monitoring team would debrief the investigators on the key issues identified as well as recommend appropriate action for those issues that required immediate action as the site awaited the final report.

## Discussion

The model employed by the site monitoring team in Uganda appears to be quite comprehensive and effective in identifying many aspects of best research practices as well as non-compliance at the research sites. The findings of this review reveal that research protocol non-compliance is still a problem in the country. To address this problem, research site monitoring should be intensified, and important aspects should be evaluated including; regulatory documents, site facilities, informed consent process and documentation, participant welfare, SAE management and reporting, study related training and working practices at the study site. Research ethics aspects were found wanting in many situations and this needs to be improved on.

Concerning regulatory issues, the reviewed reports emphasized the fact that non-compliance by investigators is an important issue to consider. On site compliance monitoring could be the most appropriate method to minimize such non-compliance, but is often ignored by the RECs purportedly due to lack of capacity, requires high cost to maintain both in terms of human resource as well as financial resources.

Although Uganda has 14 accredited RECs that review and approve research currently, only four have reported carrying out monitoring of approved studies at the site and only one was found to have records at the UNCST as evidence for conducting on site compliance monitoring (Table 2). But the current process of accreditation which is ongoing is expected to set standards and improve on the functionality of many RECs so that effective execution of their mandates are incorporated in the day to day business. And when such accreditation is accomplished, it will be a positive challenge to such RECs which have in the past grossly ignored continuing review including compliance monitoring as an essential component of their obligations.

**Table 2:** Regulatory agencies and sites reviewed

**Regulatory agency**	**Number of sites**	**Percentage sites**	**Number of projects**	**Percentage projects**
UNCST	18	64.28%	30	75%
UNCST/NDA	5	17.86%	5	12.5%
NARC	5	17.86%	5	12.5%
**Total**	**28**	**100%**	**40**	**100%**

Adequacy of site facilities was relatively good though a good number had issues of congestion due to limited space. However, sites should improve skills of study staff to make them more competent at their work stations. Investigators should also be in touch with their study sites and regularly conduct internal auditing.

The informed consent process is an important component of ethical research that may not be adequately understood and or practiced by many investigators. And this echo’s previous studies [14]. Proactive approaches like sensitization, continuing education and outreach to researchers and other stakeholders in research could yield better outcomes than strict punitive treatment and humiliation.

Research participants may be reimbursed for lost earnings, travel costs, lunch and other expenses incurred in taking part in a study; they may also receive free medical services. Research participants**,** particularly those who receive no direct benefit from the research project will be compensated for inconvenience and time spent. The compensation or medical services shall not be out of proportion so as to induce prospective research participants to consent to participate in the research against their better judgment [1,4]. Investigators risk compromising the participant welfare if they (investigators) falsely think that that such facilitation to participants could result in undue inducement and or coercion. It is better for a competent REC to decide on amount of facilitation acceptable and will not appear to coerce the participants. Participants should not bear the burden of participating in research as well as paying for expenses related to their participation in a study. Investigators and institutional review boards make payment decisions and that both healthy and ill subjects in some studies are paid for their time, for inconvenience, for travel, as incentive, or for incurring risk. Most organizations require that payment be prorated and described in the consent document [19]. It should be noted that in this review, many of the research participants were not well facilitated due to lack of due consideration for their compensation and welfare by the RECs at the time of approval of the study.

None reporting of SAEs and reporting such SAEs inappropriately are violations of what should be considered good clinical practice. Ideally, no clinical trials should be initiated unless all the study staff have undergone training in good clinical practice and where appropriate good laboratory practice in addition to being equipped with an adequate SAE management and reporting plan. The Investigator undertakes prime responsibility for monitoring and reporting of adverse events to the regulatory authorities. (1) Both investigators and regulatory agencies should ensure that study staff access GCP and related training prior to initiation of the study. SOPs must be present and accessible at each duty station and should be adhered to. In this review, the high rate of non-reporting of such SAEs is a reminder that research ethics and GCP trainings are mandated, should be appreciated and practiced.

In Uganda based on records available at the UNCST, about 50 research projects had been monitored over the period 2007 to 2010 though only three regulatory agencies had actually conducted the visits. Compliance monitoring should be scaled up and taken up by RECs because it is feasible even in a low resource setting. Doing so would be a great break through which would enhance the protection of the rights and welfare of human research participants and promote ethical conduct of research.

## Conclusion

This model employed by the monitoring teams in Uganda to evaluate research compliance appears to be effective in auditing ethical practice and if implemented appropriately can lead to improved ethical conduct by researchers as well as improved protection of the rights and welfare of human research participants.

Compliance monitoring is feasible and affordable in a resource limited setting as evidenced by this review.

Research protocol non compliance is still a major problem in Uganda, and there is need for a pro-active approach to this vice by all stake holders including research regulators, community leaders, political leaders and researchers if ethical conduct of research is to be achieved. Only competent RECs with compliance monitoring plans in their activities should be allowed to review and approve research studies.

Knowledge for both local and international regulations guiding the conduct of research is still largely ignored by many researchers with resultant violation of the approved protocols. As a policy, all study staff should undergo mandatory training in the relevant guidelines before ever getting in contact with any study participants.

### Limitations of the study

•The relatively small number of reports reviewed.

•Some reports were incomplete hence not included in the study.

•Lack of adequate detail in some of the reports because the monitors were different and also the reasons for monitoring in some cases were different.

## Competing interest

The authors declare that they have no competing interests.

## Authors’ contribution

JO Literature search, study design, data collection, data analysis, data interpretation, drafting, writing, proof reading and approval of manuscript; JE study design, data collection, data analysis, proof reading and approval of manuscript; FN study design, proof reading and approval; PK proof reading and approval of manuscript. All authors read and approved the final manuscript.

## Pre-publication history

The pre-publication history for this paper can be accessed here:

http://www.biomedcentral.com/1472-6939/14/23/prepub
